# PRAWNS: compact pan-genomic features for whole-genome population genomics

**DOI:** 10.1093/bioinformatics/btac844

**Published:** 2022-12-29

**Authors:** Kiran Javkar, Hugh Rand, Errol Strain, Mihai Pop

**Affiliations:** Department of Computer Science, University of Maryland, College Park, MD 20742, USA; Joint Institute for Food Safety and Applied Nutrition, University of Maryland, College Park, MD 20740, USA; Center for Food Safety and Applied Nutrition, United States Food and Drug Administration, College Park, MD 20740, USA; Center for Veterinary Medicine, United States Food and Drug Administration, Laurel, MD 20708, USA; Department of Computer Science, University of Maryland, College Park, MD 20742, USA

## Abstract

**Motivation:**

Scientists seeking to understand the genomic basis of bacterial phenotypes, such as antibiotic resistance, today have access to an unprecedented number of complete and nearly complete genomes. Making sense of these data requires computational tools able to perform multiple-genome comparisons efficiently, yet currently available tools cannot scale beyond several tens of genomes.

**Results:**

We describe *PRAWNS*, an efficient and scalable tool for multiple-genome analysis. *PRAWNS* defines a concise set of genomic features (metablocks), as well as pairwise relationships between them, which can be used as a basis for large-scale genotype–phenotype association studies. We demonstrate the effectiveness of *PRAWNS* by identifying genomic regions associated with antibiotic resistance in *Acinetobacter baumannii*.

**Availability and implementation:**

*PRAWNS* is implemented in C++ and Python3, licensed under the GPLv3 license, and freely downloadable from GitHub (https://github.com/KiranJavkar/PRAWNS.git).

**Supplementary information:**

Supplementary data are available at *Bioinformatics* online.

## 1 Introduction

Rapid advances in sequencing technology have led to the availability of genomes for many bacteria, viruses and fungi. The study of these genomes has been crucial in improving our understanding of different organisms; it is transforming public health and food safety monitoring, and has led to the development of sequence-based infectious disease programs (Armstrong *et al.*, 2019; Timme *et al.*, 2018). Methods developed for genomic analysis of small numbers of bacterial genomes, such as the alignment, gene-based and *k*-mer approaches described in more detail below, are not able to keep pace with the analysis demands of an ever-increasing number of isolates produced through public health surveillance.

Genomic comparisons provide valuable insights into the evolutionary biology of organisms and allow the characterization of variation by, e.g. horizontal gene transfer events, insertions–deletions (indels) and translocations. Such genomic variations can cause important phenotypic variations, including antimicrobial resistance (AMR) and pathogenic variation (Boyd *et al.*, 2009). Analysis of genomic variations benefit from a pan-genome representation—genomic features present across a set of genomes are aggregated into a single unified representation (Tettelin *et al.*, 2005), which readily permits assessing the genomic features potentially influencing phenotypic variation (Sheppard *et al.*, 2018). Large-scale genome comparisons (also referred to as population genomics or comparative genomics) permit the association of such genomic variants with the phenotypic variation due to geography, environment, AMR and as found in case–control studies (Worley *et al.*, 2021). The understanding of relevant genomic factors and mechanisms underlying phenotypic variation enhances our ability to monitor phenotypes in important applications, such as microbial food safety, disease invasiveness and AMR (Halpin *et al.*, 2021).

Comparison of multiple closely related genomes can be performed in a number of ways. The most obvious one is via whole-genome alignment, and well-known tools for this include Mauve (Darling *et al.*, 2004), Mugsy (Angiuoli and Salzberg, 2011) and Cactus (Paten *et al.*, 2011). Another approach is gene-focused and uses gene prediction followed by gene clustering to estimate the core and accessory genes. Prominent tools include PanSeq (Laing *et al.*, 2010), PGAP (Zhao *et al.*, 2012) and Roary (Page *et al.*, 2015). Other faster approaches rely on exact matches to construct a colored de Bruijn graph (cdBg): these tools, such as SplitMEM (Marcus *et al.*, 2014), TwoPaCo (Minkin *et al.*, 2017), locate exact-matching substrings of fixed length—called *k*-mers—from all genomes and aggregated them into a compacted graphical representation for all genomes. A recently developed multiple whole-genome alignment pipeline, SibeliaZ (Minkin and Medvedev, 2020), uses TwoPaCo to construct a de Bruijn graph, followed by locally collinear blocks (LCBs) extraction with SibeliaZ-LCB, and finally running spoa for multiple sequence alignment (Vaser *et al.*, 2017). Among the above approaches, the compacted de Bruijn graph-based ones generally scale to large numbers of genomes without limiting themselves to variants only present in genes.

Recently, some other approaches, called bacterial genome-wide association studies (GWAS), have been developed specifically for genotype–phenotype correlation studies; prominent ones include SEER (Lees *et al.*, 2016), DBGWAS (Jaillard *et al.*, 2018). These approaches start by extracting *k*-mers from the genomes or sequenced reads. These *k*-mers or the exact matches from compacted de Bruijn graphs constitute the key features in any subsequent analysis of the genomes. Each of these methods is fast but loses some of the genomic context information that may potentially impact downstream analysis results.

All approaches discussed in the last two paragraphs have their strengths and weaknesses. The methods based on whole-genome alignment do not scale adequately with current sequencing capacity (>100 genomes). Reference-guided approaches [e.g. Davis *et al.* (2015)] suffer from the inherent bias in the reference chosen as well as the need for a closely related reference genome; species with an ‘open’ pan-genome are a poor fit for a reference-based approach. Gene-based approaches handle the entire pan genome, but suffer from gene prediction biases and gene clustering thresholds (Doyle *et al.*, 2020); additionally, poor assembly quality of the genomes is problematic for the gene prediction process. Compacted de Bruijn graph approaches are fast, scalable and not limited to genes. However, the graph output comprises a large number of exact matches (‘unitigs’), which makes the downstream analysis difficult. None of the current approaches provide a full solution to the problem of providing a well-scaling algorithm that provides genomic context and supports GWAS easily.

Variation in genomic context of specific genetic features can drive phenotypic variation. However, such variation has been largely understudied due to the inability of existing genome comparison approaches to support an efficient comparative assessment of the genomic context. As such, analyses of genomic context have largely been limited to the relationship between genes and the changes in their promoter regions.

Promoter region changes can influence the expression levels of corresponding genes—an important AMR mechanism. Certain AMR genes are upregulated due to the presence of insertion sequences, a type of mobile element, in their promoter regions (Evans *et al.*, 2013). Mobile elements are known to pose challenges to genome assembly algorithms and often get assembled in separate contigs in draft assemblies (Adams *et al.*, 2016; Javkar *et al.*, 2021). The fragmentation in draft assemblies prohibits a comprehensive comparison of multiple genomes while accounting for such collocated occurrences of genomic factors, which may be influential on the resultant phenotype. Overall, for a thorough comparative analysis of several related genomes, we need an approach that is scalable, accounts for the variations at a whole-genome level, and provides a reasonably small number of genomic features, which could be examined for a downstream assessment.

Here, we propose a novel approach, *PRAWNS*: a fast and scalable tool that generates an efficient representation of closely related whole genomes to provide a concise list of genomic features or sequence entities shared by a user-specified fraction of the genomes. *PRAWNS* can be parallelized over multiple threads and uses disk-based storage, enabling it to scale to thousands of genomes. *PRAWNS* relies on two main algorithmic innovations. First, we identify segments of DNA that are shared across the genomes being analyzed; we call these segments the ‘conserved regions’. The detection of conserved regions begins with the identification of exact-matching regions, which are later merged into ‘metablocks’. These metablocks may contain inexact matches, and reduce the number of features to be considered by an order of magnitude over individual exact-matching blocks. *PRAWNS* achieves efficiency by using exact matches and by organizing them into metablocks without the need of an actual alignment. Our second algorithmic contribution is a new type of genomic feature called ‘paired regions’—these are pairs of conserved regions collocated in multiple genomes but that may vary in distance between each other within different genomes. Using such paired features, scientists can assess the influence of the collocation of genomic segments on phenotypes.

## 2 Materials and methods

### 2.1 Overview, notation and definitions

*PRAWNS* takes as input a set of whole genomes $G=\{G_{1},\ldots ,G_{\left|G\right|}\}$, where |G| denotes the total number of input genomes. Optionally, it also supports contig orientations obtained from using scaffolding (Ghurye *et al.*, 2019). For each genome, we find the *k*-mers that are unique in it; these *k*-mers are then merged to get shared exact-matching regions, referred to as ‘blocks’. Note that the blocks are similar to the maximal unique matches used in many other genome alignment approaches (Delcher *et al.*, 1999), except that they need not be maximal in each genome that contains them. Next, we determine the ‘neighborhood’ for each block—a block is defined to be in the neighborhood of another block if the separation between these blocks is at most a threshold *δ* nucleotides in some genome *G_i_*; such a pair of blocks is called a ‘candidate neighboring pair’. We employ these candidate neighboring pairs to identify the clusters of collocated blocks, referred to as the ‘components’ of collocated blocks shared across multiple genomes (Algorithm 1). The collocated blocks from each component are merged to generate ‘metablocks’ (Algorithm 2): each metablock is a chain of blocks that are present in the same order in at least a user-defined fraction (*ϵ*) of genomes under study. Additionally, we provide a set of ‘retained blocks’ comprising the blocks, which were not merged into a metablock but are at least *γ* nucleotides long. The metablocks and retained blocks together constitute the primary set of *PRAWNS*’ features, and are referred to as the ‘conserved regions’—denoting the genomic regions shared between multiple genomes under study. We avail the conserved regions to compute an additional set of contextual genomic features—the paired regions. A paired region denotes a pair of conserved regions that are closely adjacent (≤Δ) with a consistent relative orientation in ≥ϵ×|G| genomes. All aforementioned thresholds are user-defined and their default values are provided in the subsequent sections.

We use a single coordinate system per genome: genomic coordinates start at 1, and increase from left to right. Contigs within an assembled genome are concatenated while maintaining the contig boundaries. For example, if the first contig is 1000 bp long, the next contig would start at 1001. A genomic feature *f*, i.e. a block or a metablock, located in genome *G_i_* is represented by the tuple $\langle f.\mathrm{star}t_{i},f.end_{i},f.\mathrm{orientatio}n_{i}\rangle$: start_*i*_ and end_*i*_ are the left-most and right-most coordinates of *f* in *G_i_*, while orientation_*i*_ is 1 or 0 if *f* is located on the forward or reverse strand, respectively, in *G_i_*. A start_*i*_ =0 indicates that *f* is missing in *G_i_*. Two features (metablocks or retained blocks) *f_a_* and *f_b_* constitute a paired region *pr* if they are closely adjacent (≤Δ) with a consistent relative orientation in ≥ϵ×|G| genomes. If *pr* exists in a genome *G_i_*, then the value for *pr* in *G_i_* denotes the nucleotide distance separation between *f_a_* and *f_b_* in *G_i_*. If *pr* does not exist in *G_i_*, then the corresponding value is 0.

### 2.2 Conserved regions detection

#### 2.2.1 Exact-matching blocks

We begin with detecting *k*-mers (*k* is user-defined, default: 25) from the given genomes—the idea is to find maximal exact-matching regions shared across multiple genomes. We only consider unique *k*-mers from each genome, i.e. *k*-mers present only once within that genome, and construct a cdBg representation: a vertex denotes a unique *k*-mer, an edge exists in the graph if the corresponding (k+1)-mer formed using the adjoining two *k*-mers exists in the input genomes, and the edge-color denotes the genome membership for that (k+1)-mer. The cdBg is pruned to remove the vertices and edges that are present in fewer than ϵ×|G| genomes, where *ϵ* (default value: 0.05) can be user-specified. The pruned graph is ‘compacted’: a path *P* is compacted into a single vertex if all its vertices barring the first vertex have an in-degree 1, all but the last vertex have an out-degree 1 and each edge e∈P has an identical set of edge-colors. Thus, each compacted vertex represents the corresponding exact-matching region present in ≥ϵ×|G| genomes—the exact-matching regions are referred to as ‘blocks’, denoted by **B**. The blocks present in genome *G_i_* are indicated by *B_i_*.

Existing cdBg-based pan-genome tools typically stop at graph compaction and output the vertices and edges. Even with a small number, say 50, of a bacterial species’ genomes, the vertex count usually exceeds 10^5^. Statistical analysis directly using these features would assume them to be independent. However, we observe the blocks to be often collocated in multiple genomes, i.e. in linkage disequilibrium. It is desirable to eliminate this dependency between the features and improve their statistical analyses. We remove this dependency by representing the collocated blocks into a single aggregated feature; this is performed over two phases: (i) identifying ‘components’ of collocated blocks and (ii) locating ‘metablocks’ from these components (see Supplementary Section S1).

#### 2.2.2 *Components* of collocated blocks

**Fig. 1.**
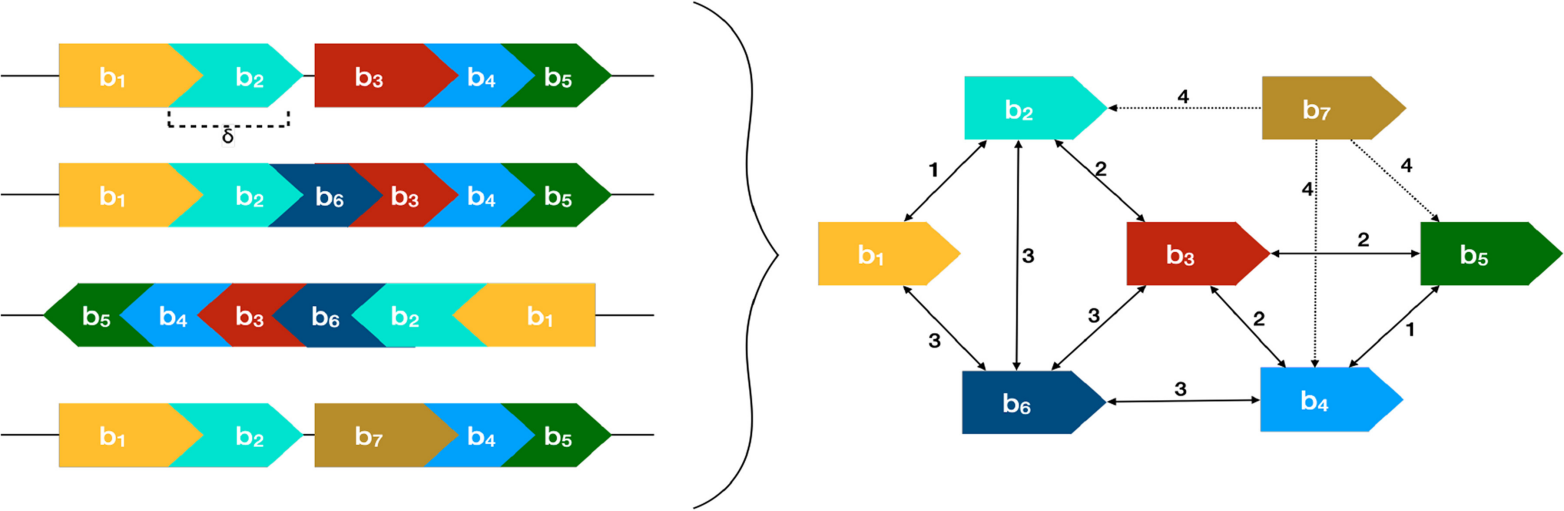
*K*NN graph DG (right) constructed from the blocks identified from four genomes (left) using the Algorithm 1. *K *=* *4 and φ=0.5. Blocks collocated ≤δ nt apart in some genome/s represent the candidate neighbors. The weight of an edge *e*(*u*, *v*) is given by $w(u,v)=|G|-|G_{u,v}|+1$; $\left|G_{u,v}\right|$ signifies the number of genomes, where *v* is a candidate neighbor of *u*. For example, blocks *b*_2_ and *b*_4_ are candidate neighbors in two of the four genomes, so $w(b_{2},b_{4})$=4−2 + 1 = 3. The dotted edges (unidirected neighborhood) are discarded to get

Several blocks are often located within a proximity of one another across multiple genomes. We formulate the detection of such collocated blocks via construction of a *K*-nearest neighbors (KNN) graph and identifying the connected components in this graph (Fig. 1 and Algorithm 1). Here, we make use of the fact that the genomes are linear (or circular): for each block, we can determine the blocks located within its ‘neighborhood’ on each genome. A block *v* is defined to be a candidate neighbor of another block *u* if the separation between these blocks is at most a threshold *δ* (user-defined, default: 5) nucleotides in some genome *G_i_*; such a pair of blocks (*u*, *v*) is called a ‘candidate neighbor pair’. For a candidate neighbor pair (*u*, *v*), $\left|G_{u,v}\right|$ denotes the number of genomes in which this candidate neighbor pair exists and is referred to as its genome occurrence count. For a block *u*, if $\left|G_{u,v}\right|$ has the highest genome occurrence count among all candidate neighbor pairs with *u*, then another candidate neighbor *t* is deemed a frequently encountered candidate neighbor of *u* if $\left|G_{u,t}|\geq (1-\varphi )\times |G_{u,v}\right|$, where φ (default: 0.05) can be user-specified (see Supplementary Section S2). For example, in Figure 1, |G|=4 and φ=0.5: for blocks *b_i_* and *b_j_*, *b_j_* would be a frequently encountered candidate neighbor of *b_i_*, if *b_j_* is a candidate neighbor of *b_i_* in at least ((1−0.5)×4=) two genomes. Using these candidate neighbor pairs, we create a *K*NN graph: the vertices represent the blocks and each block has an edge directed toward at most *K* blocks corresponding to the blocks that formed the *K*-most frequently encountered candidate neighbor pairs. *K* is chosen to be a small integer and dependent on *δ* $\left(K\approx \sqrt{\delta }\right)$; this ensures that the graph is sparse but collocated blocks remain connected. Ties are broken arbitrarily in cases with more than *K* neighboring blocks. For an edge *e*(*u*, *v*), its edge weight $w(u,v)=|G|-|G_{u,v}|+1$, i.e. the most frequently encountered candidate neighbors have the lowest edge weights. The *K*NN graph is pruned to retain the reciprocal nearest neighbors: *u* and *v* are reciprocal nearest neighbors if both *u* and *v* are *K*NN of each other, i.e. both the edges *e*(*u*, *v*) and *e*(*v*, *u*) exist in the *K*NN graph. Connected components are then extracted from the resultant graph UG and are referred to as the ‘components’ of collocated blocks, denoted by **C**. The run-time complexity of Algorithm 1 is O(|G||B| log(|B|)).

Algorithm 1: Algorithm for determining the components of collocated blocks

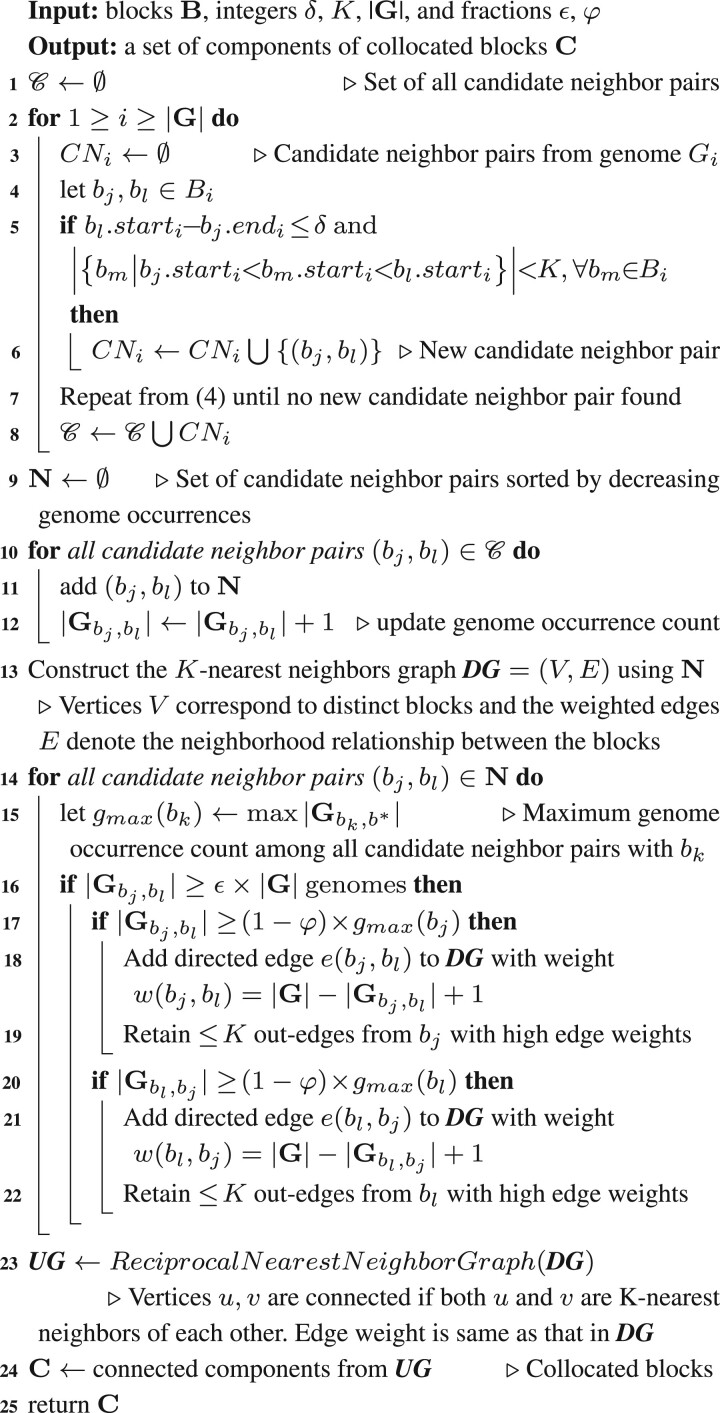

#### 2.2.3 Metablocks

‘Metablocks’ are chains of blocks that are present in the same order in ≥ϵ×|G| genomes. Algorithm 2 describes the process to identify the metablocks from the components of collocated blocks. First, we determine the genomes, which are likely to encode the metablocks by ascertaining the genome membership of their corresponding components: for each component *c*, we compute the number of blocks from *c* that are present within each genome. A component is considered to be present in a genome *G_i_* if at least *ω_c_* of its blocks (by default, 90%) are present in *G_i_*. The corresponding subset of genomes containing the component *c* is denoted as $\tilde{G_{c}}$. Next, to ensure a consistent ordering of blocks within a metablock region, we identify the ‘core block pairs’ for each component: blocks *u* and *v* form a core block pair of component *c*, if *u* and *v* are adjoining pairs of blocks with a consistent relative orientation in each genome $G_{i}\in \tilde{G_{c}}$. The core block pairs are a subset of candidate neighboring blocks. By design, adjacent core block pairs can have at most one block in common; core block pairs that share a block are extended to form ‘chains’. Chains from a component *c* are merged and extended, if they are at most a certain distance, *μ* (user-defined, default: 25), apart and have a consistent relative orientation in each genome $G_{i}\in \tilde{G_{c}}$—the resultant merged region constitutes a ‘metablock’ (see Supplementary Section S2). A larger *μ* can yield longer metablocks with several mismatches corresponding to the substitutions and indels between adjacent chains. If contig orientations (via scaffolding) are provided, chains can also be merged to generate composite metablocks that span across contig boundaries. Algorithm 2 has a run-time complexity of O(|G||B|).

Algorithm 2: Algorithm for constructing metablocks from components of collocated blocks

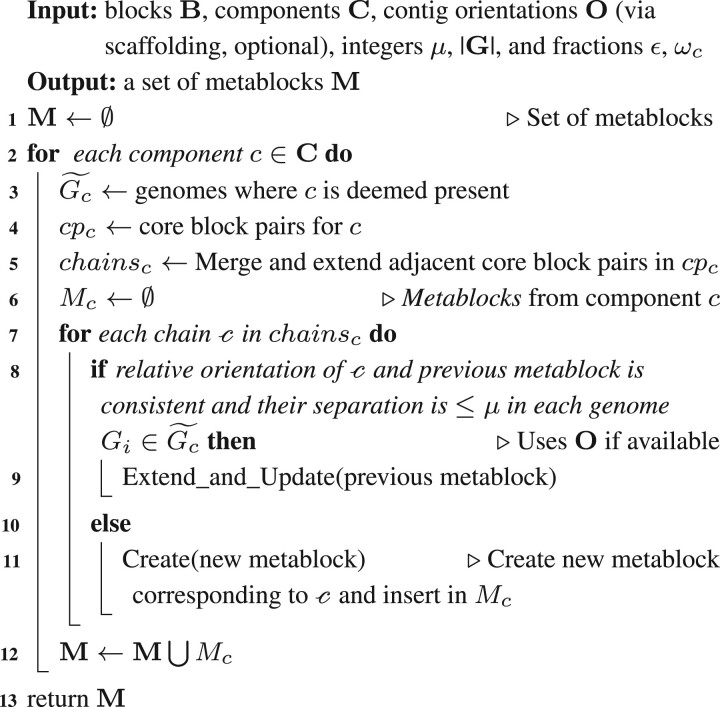

Empirically, we observe that many components comprise just one or two blocks, potentially originating from spurious *k*-mers (see Supplementary Section S4). We, therefore, confine the metablock detection to the components with at least three blocks to get longer metablocks and remove noise. Observe that each block can be a part of only one component and can, hence, contribute to at most one metablock. However, some blocks may not be merged into a metablock either because they formed components with at most just one more block or the block was present but the corresponding component was not deemed present in certain genomes. Unmerged blocks, longer than a threshold *γ* (user-defined, default: 50), are referred to as ‘retained blocks’; the metablocks and the retained blocks together comprise the ‘conserved regions’—the primary set of *PRAWNS*’ pan-genome features. The conserved regions output constitute their FASTA sequences and two tabulated files denoting the genomic coordinates and binary presence/absence in respective genomes.

### 2.3 Paired regions detection

Identical genomic regions can give rise to different phenotypes depending on their orientation and association with other genomic regions (Evans *et al.*, 2013; Jiang *et al.*, 2019). To capture such paired interactions, we developed the ‘paired regions’ feature in *PRAWNS*: each feature corresponds to two conserved regions, *r*_1_ and *r*_2_, and their relative orientations such that *r*_1_ and *r*_2_ are separated by at most a small distance Δ (user-defined, default: 50) nucleotides in ≥ϵ×|G| genomes. Note that for a pair of conserved regions *r*_1_ and *r*_2_, a feature is constructed for each distinct pair of relative orientations. Once the paired regions are identified, they are scanned across the other genomes to check if they are present (same orientation) but farther apart (>Δ nucleotide separation).

The output for the paired regions is represented by two comma-separated files: one file provides its binary presence/absence in each input genome while the other contains the nucleotide separation between the genomic coordinates of the constituent conserved regions. A negative value for separation corresponds to overlap between the conserved regions in the respective genomes. Observe that the output file containing the separations also allows assessing whether the distance between conserved regions is influential in the downstream assessments.

### 2.4 Implementation

*PRAWNS* is an open-source code available under the GPLv3 license at https://github.com/KiranJavkar/PRAWNS.git. It is implemented in C++ and Python3 and works on Unix-like operating systems. The default parameters are calibrated for bacterial genomes. Detailed documentation for *PRAWNS* is available along with its source code.

*PRAWNS* is designed to work using limited main memory (RAM usage) with the availability of disk usage and supports parallelization. Each module of the tool—identification of blocks, components of collocated blocks (Algorithm 1), metablocks (Algorithm 2) and paired regions—can be executed in parallel over *tc* cores (user-defined, default: 8) that access a shared disk space. If the maximum length of given genomes is *N* and the total sequence length is *n*, then the run-time complexity to detect the pan-genome features is O(n log N+|G||B| log(|B|)). For closely related genomes with roughly similar genome lengths (n≈|G|×N), the run-time is linear in the number of input genomes.

### 2.5 Analysis

#### 2.5.1 Genome assembly

Sequenced reads for all genomes analyzed were downloaded from NCBI and assembled with SPAdes (v3.13.0, default settings) (Bankevich *et al.*, 2012). In order to ensure better confidence in the genomic variants detected within these assembled genomes, the genomes were filtered to retain only those contigs that had at least 10× sequence coverage.

#### 2.5.2 Statistical significance testing

It is important to note that the statistical analysis described here is simply meant to demonstrate the use of the features generated by *PRAWNS*, and is not an integral part of *PRAWNS* itself. The exploration of different statistical approaches for conducting association studies based on *PRAWNS* features is beyond the scope of this manuscript. For genotype–phenotype correlation analysis, the statistical significance of the genomic features was assessed using Fisher’s exact test and the Benjamini–Hochberg procedure for false discovery rate (FDR) correction (α=0.01). A feature was deemed to be statistically significantly correlated with the phenotype of interest if it had an odds ratio ≥2 and an FDR-adjusted *P*-value ≤0.01.

## 3 Results

### 3.1 Datasets

We benchmark the performance of *PRAWNS* on a whole-genome dataset of 362 *Acinetobacter baumannii* isolates (bacteria, ∼4 Mbp per genome) for which experimentally validated imipenem-resistance information was available (Javkar *et al.*, 2021). To assess the scalability, *PRAWNS* was executed on two additional datasets: (i) 4000 *Salmonella* Infantis genomes (bacteria, ∼5 Mbp per genome) and (ii) 107 *Aspergillus flavus* genomes (fungi, ∼38 Mbp per genome) (Supplementary Table S1).

### 3.2 Methods compared

As of the time of writing this manuscript, only SibeliaZ-LCB (Minkin and Medvedev, 2020) is able to generate a pan-genome representation at a whole-genome scale, can handle a large number of genomes, and processes a compacted de Bruijn graph to produce a reduced set of genomic features—the LCBs. As the LCBs defined by SibeliaZ-LCB are similar to the conserved regions from *PRAWNS*, we compared the performance of *PRAWNS* against SibeliaZ-LCB; we limit the multiple whole-genome alignment pipeline SibeliaZ to only the first two steps, i.e. compacted de Bruijn graph construction using TwoPaCo followed by LCB detection with SibeliaZ-LCB. However, it is important to note that SibeliaZ-LCB is intended for detection of homologous sequences that have an evolutionary distance to the most recent common ancestor of at most 0.09 substitutions per site, and does not account for potential rearrangements or larger structural changes.

We used the combination of TwoPaCo (v0.9.3) and SibeliaZ-LCB (v1.2.2) (hereafter referred to as TwoPaCo+SibeliaZ-LCB) for comparative benchmarking of *PRAWNS*’ performance. TwoPaCo was run with filtersize10. SibeliaZ-LCB was run with the parameters -m50-b25 to maintain consistency with the default values used for *PRAWNS* parameter ϵ,γ,Δ. DBGWAS was run with default parameters and *k *=* *25 (Jaillard *et al.*, 2018). All tools were run on a 64 core Xeon E5-2680 server running at 2.70 GHz and a total of 256 GB of RAM.

### 3.3 Performance

#### 3.3.1 Scalability

**Fig. 2.**
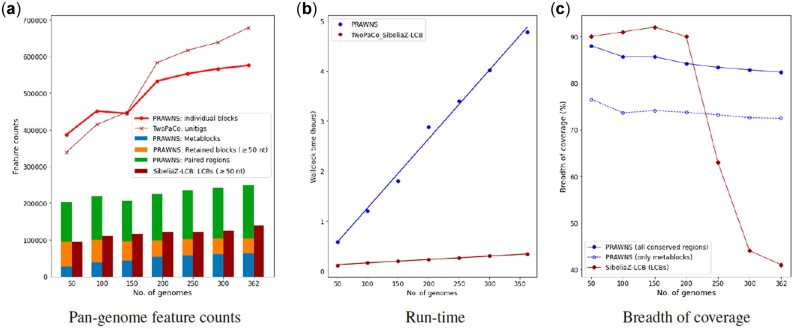
Scalability performance using *A.baumannii* dataset. (**a**) Feature counts from *PRAWNS* and SibeliaZ-LCB. For comparison, the number of blocks (*PRAWNS*) and unitigs (TwoPaCo) are shown. The total number of conserved regions from *PRAWNS* is the combination of metablocks and the retained blocks (*γ *= 50). (**b**) Run-time performance of *PRAWNS* and SibeliaZ-LCB. (**c**) Median breadth of coverage by the conserved regions and LCBs

Figure 2 compares the performance and feature counts on the *A.baumannii* dataset. The dataset (362 isolates) was randomly sampled without replacement to create smaller datasets of 50, 100, 150, 200, 250 and 300 genomes. The tools were run with *k*-mer length 25 on 8 cores and the maximum memory usage was limited to 36 GB. Figure 2a shows line plots for the counts of exact-matching regions detected in the genomes: blocks from *PRAWNS* (dense red line) and unitigs from TwoPaCo (thin red line with ×’s). The bar plots represent the feature counts generated by *PRAWNS* and SibeliaZ-LCB. The merger of blocks into metablocks (blue bars) results in an order of magnitude reduction in their counts. The total counts of conserved regions, i.e. metablocks and retained blocks (orange bars), are comparable to that of the LCBs from SibeliaZ-LCB (maroon bars). Observe that the total counts of *PRAWNS*’ features (conserved regions and paired regions) are still much smaller than that from mere de Bruijn graph compaction. Figure 2b shows that both—*PRAWNS* and TwoPaCo+SibeliaZ-LCB—can operate on large number of closely related whole genomes with a run-time linear in the number of input genomes, but TwoPaCo+SibeliaZ-LCB is much faster than *PRAWNS*. The actual RAM usage for *PRAWNS* was under 8.3 GB while that for TwoPaCo+SibeliaZ-LCB was under 3 GB for the complete set of 362 genomes. Figure 2c demonstrates the breadth of coverages for the genomes obtained using conserved regions from *PRAWNS* and LCBs from SibeliaZ-LCB. Here, we see a stark difference between the features obtained from the two approaches. The median breadth of coverage with *PRAWNS*’ conserved regions is 88% for 50 *A.baumannii* genomes and slightly reduces with added genomes, reaching 82% for all 362 genomes. The LCBs from SibeliaZ-LCB account for a median genome coverage of 90% for 50 genomes; the LCBs from SibeliaZ-LCB account for a median breadth of coverage of 90% for 50 genomes; however, on larger datasets, the breadth of coverage drops rapidly and reached 41% using all 362 genomes.

**Fig. 3.**
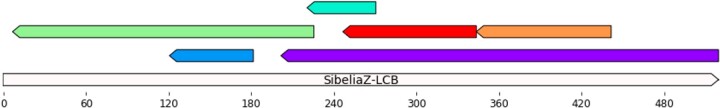
Conserved regions from *PRAWNS* aligned to a 520 nt LCB from SibeliaZ-LCB. The two longer conserved regions are metablocks while the remaining four are additionally retained blocks (*γ *= 50). The unique color assigned to each conserved region signifies distinct *presence vectors*, i.e. different genome memberships. SibeliaZ-LCB presumes this to be a contiguous homologous region and be deemed present or absent in the genomes

Next, we explore the similarities and differences between the conserved regions and LCBs using the output for all 362 genomes. The LCBs were mapped to conserved regions using BLAST. With at least 90% identity, 97 612 out of 102 623 (95.12%) conserved regions mapped to 113 527 out of 139 968 (81.11%) LCBs. However, multiple conserved regions mapped to a single LCB and simultaneously also mapped to multiple LCBs—suggesting redundancy in the genomic regions marked by different LCBs. Figure 3 shows one such example where SibeliaZ-LCB identified a single LCB of 520 nt length, whereas *PRAWNS* decomposed this region into six conserved regions; the two longer ones are metablocks. The colors of these conserved regions denote the *presence vectors* (or equivalence classes or color classes) for these regions. We observe that these regions have different genome memberships and, therefore, should not be represented as a single collinear homologous region.

To demonstrate *PRAWNS*’ scalability to thousands of genomes, we ran it on 4000 *Salmonella enterica* subsp. *enterica* serovar Infantis (*S*. Infantis)—all isolates were selected from an NCBI Pathogens SNP cluster (cluster ID: PDS000089910, Supplementary Table S1) and had a mean total genome length of 4.97 Mbp. *PRAWNS* was executed using default parameters on 16 cores each of 2.30 GHz Xeon E5-2650 processor with 50 GB maximum RAM limit. The execution completed in 24 h (24.09 GB peak memory usage) and generated a pan-genome comprising 55 366 conserved regions (6553 metablocks and 48 813 retained blocks), yielding a median breadth of coverage of 89.41%, and 86 175 paired regions.

To assess the scalability of *PRAWNS* on longer (eukaryotic) genomes, we ran it on a fungal dataset comprising 107 *A.flavus* genomes downloaded from NCBI (see Supplementary Section S3). *Aspergillus flavus* contains eight chromosomes and the genomes are ∼38 Mbp each. Using the default *k*-mer length (25) on five cores each of 2.70 GHz Xeon E5-2680 processor with 50 GB maximum RAM usage limit, *PRAWNS* required 20 h (40.26 GB peak memory usage) to generate the pan-genome comprising 858 081 conserved regions [427 698 metablocks and 430 383 retained blocks (γ=50)] and 1 020 418 paired regions (Δ = 50). As in the case of *A.baumannii* genomes, the run-time was linearly proportional to the number of *A.flavus* genomes.

#### 3.3.2 Robustness to the choice of *k*-mer length

**Fig. 4.**
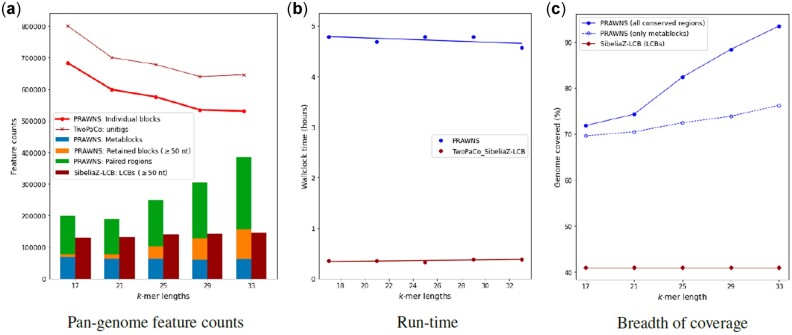
Impact of *k*-mer length using *A.baumannii* dataset (362 genomes). (**a**) Feature counts from *PRAWNS* and SibeliaZ-LCB. The number of blocks and unitigs are shown for comparison. The total number of conserved regions from *PRAWNS* is the combination of metablocks and the retained blocks (*γ *= 50). (**b**) Run-time performance of *PRAWNS* and SibeliaZ-LCB. (**c**) Median breadth of coverage by the conserved regions and LCBs

Figure 4 shows the influence of choice of *k*-mer length on the entire *A.baumannii* dataset. Figure 4a shows a steady decrease in the number of blocks with an increased choice of *k*-mer length. However, the number of metablocks remains largely the same irrespective of the *k*-mer length. The *k*-mer length, however, varies the counts for the longer retained blocks and paired regions: smaller *k*-mer length yields fewer longer blocks and paired regions whereas larger *k*-mer length results in more longer blocks and paired regions. The LCB counts from SibeliaZ-LCB remain fairly unchanged by the *k*-mer length choice. As observed in Figure 4b, the run-time performances of both, *PRAWNS* and TwoPaCo+SibeliaZ-LCB, are robust to the choice of *k*. The actual RAM usage was consistent with before for all these configurations. Figure 4c shows the impact of *k*-mer lengths on the median breadth of coverage of the genomes by resultant conserved regions and LCBs. The increase in *k*-mer lengths contributes to higher breadth of coverage at the expense of more conserved regions that would need to be analyzed. The breadth of coverage by metablocks alone increased slightly from 70% to 76% with an increase in *k*-mer length from 17 to 33. The overall increase in the breadth of coverage can be attributed to the detection of more unique *k*-mers and longer blocks (and, hence, an increased count for the retained blocks) with an increase in *k*. In contrast, the breadth of coverage using LCBs from SibeliaZ-LCB remains at around 41%—unperturbed by *k*-mer length choice.

We assessed the precision and recall for *PRAWNS* conserved regions by taking the LCBs from Mauve (Darling *et al.*, 2004) as truth and evaluating the results on a dataset of 16 *Campylobacter jejuni* genomes from Timme *et al.* (2017). *PRAWNS* was run with default settings. The conserved regions had a high precision (≥95.2%) and high recall (≥99.8%) for the examined range of *k*-mer lengths (17–33) (Supplementary Table S2). This indicates a good agreement with the existing methods.

### 3.4 Applications

#### 3.4.1 Genotype–phenotype correlation studies

*PRAWNS* generates a pan-genome representation, which can identify the genomic features for downstream population genomic analyses. As a proof of principle, we demonstrate the utility of *PRAWNS* for population genomic studies using genotype–phenotype correlation analyses.

##### 3.4.1.1 Antimicrobial resistance (AMR) in *Acinetobacterbaumannii*

Imipenem (a type of carbapenem antibiotic)-resistance phenotypes were determined for 362 *A.baumannii* genomes using the Clinical and Laboratory Standards Institute (CLSI) antimicrobial susceptibility testing guidelines (CDC, 2017; Javkar *et al.*, 2021). Out of the 362 genomes, 305 were imipenem-resistant while the remaining 57 were imipenem-susceptible (Supplementary Table S1). The genomic features constructed with *PRAWNS* comprised 102 623 conserved regions (63 358 metablocks and 39 265 retained blocks) and 145 615 paired regions, while SibeliaZ-LCB generated 139 968 LCBs. In order to ensure consistent behavior with that of *PRAWNS*, rather than relying directly on the information provided by SibeliaZ-LCB, we aligned the LCBs to the genomes using nucmer (Marçais *et al.*, 2018) and deemed an LCB present in a genome if it aligned with ≥90% identity and ≥90% query coverage.

First, we focus on genomic features shared by almost all the genomes (core-genome features found in ≥99% genomes). The core-genome features from *PRAWNS* comprised 6641 conserved regions and 2208 paired regions. When mapped against the CARD AMR gene database (Alcock *et al.*, 2020) using BLAST, the conserved regions aligned to several resistance and virulence genes including *bla*${}_{\text{OXA}-51-\text{like}}$, *bla*${}_{\text{ADC}}$, *abeM*, *adeFGH* and *adeIJK*. The core-genome features from SibeliaZ-LCB comprised 1035 LCBs; the resistance and virulence genes aligning to these regions included *bla*${}_{\text{OXA}-51-\text{like}}$, *bla*${}_{\text{ADC}}$ and *adeIJK*.

Next, we examined the genomic features with statistically significant correlations with the imipenem-resistant phenotype (Fisher’s exact test with Benjamini–Hochberg FDR correction). The significant features from *PRAWNS* comprised 39 665 conserved regions (33 941 metablocks and 5724 retained blocks) and 75 241 paired regions, with a total sequence length of 3 Mbp, while those from SibeliaZ-LCB comprised 7424 LCBs, with a total sequence length of 7.53 Mbp. The conserved regions with significant association with resistance aligned to several AMR genes, including all 10 AMR genes [*bla*${}_{\text{OXA}-23}$, *msrE*, *mphE*, *ANT(3”)-IIa*, *aacC1*, *aphA6*, *qacEdelta1*, *sul1*, *yafP* and *xerD*] reported in our previous work (Javkar *et al.*, 2021) and known to be strongly correlated with imipenem-resistance—demonstrating the utility of *PRAWNS*’ features for such genotype–phenotype analyses. On the contrary, the significant LCBs aligned to only 2 of the 10 AMR genes strongly correlated with imipenem-resistance—*aphA6* and *xerD*—and missed many important AMR genes associated with imipenem-resistance.

In comparison with DBGWAS (*q*100) 4068 significant conserved regions were identified as ‘top’ conserved regions (significant conserved regions with among the 100 lowest FDR-adjust *P*-values). These had a total sequence length of 388 kb and aligned with five strongly correlated AMR genes: *bla*${}_{\text{OXA}-23}$, *msrE*, *qacEdelta1*, *sul1* and *xerD*. DBGWAS identified 19 subgraphs spanning a total sequence length of 175 kb and also aligned to five strongly correlated AMR genes: *bla*${}_{\text{OXA}-23}$, *msrE*, *mphE*, *ANT(3”)-IIa* and *xerD*. However, none of the significant nodes (unitigs) from these subgraphs aligned with any AMR gene.

The significant features from *PRAWNS* also support the discovery of other important and potentially understudied genomic factors influencing the phenotype. When aligned with NCBI BLAST (Johnson *et al.*, 2008), the statistically significant conserved regions also mapped to several mobile genetic elements and gene promoter regions associated with AMR, including insertion sequences (IS*Aba1*, IS*Aba13*, IS*Aba17* families), phage related genes and plasmids. Additionally, the conserved regions mapped to other genes, such as *vgrG* (Wang *et al.*, 2018) and *tviB* (Marathe *et al.*, 2019), which are relatively understudied with regard to AMR. The paired regions also provide a new dimension to the analysis: 75 241 paired regions were identified to be statistically significant, suggesting the presence of genomic factors whose co-presence is relevant to understanding antibiotic resistance.

To facilitate phylogenetic tree construction and population structure estimation for the given genomes, an auxiliary script has been provided in the *PRAWNS*’ repository. This script computes a distance matrix for the genomes using the *PRAWNS*’ output, which can be used for population structure adjustments as described, e.g. Lees *et al.* (2016).

## 4 Discussion

We developed *PRAWNS* to fill a gap in the current tool-kit available to scientists for assessing the association of bacterial genomic features with phenotypes, such as antibiotic resistance. Whole-genome alignment approaches do not scale beyond tens of hundreds of genomes, whereas the association studies relying on SNPs or *k*-mers require the assessment of features whose counts greatly exceed the number of genomes analyzed. Other studies that use genes as the features being associated with a phenotype may miss important factors, such as mutations in promoter regions and interactions between adjacent genes. With *PRAWNS*, we present a middle ground in the form of scalability to large numbers of genomes (in the manuscript, we show scalability up to 4000 genomes being aligned to each other) while maintaining a modest collection of features that facilitate the analysis of multi-chromosome isolates, allow for proper treatment of mobile genetic elements and support the assessment of draft assemblies. Additionally, the provision to analyze the interactions between genomic regions can enable uncovering biologically important genomic factors, which have been rarely characterized by multiple-genome association tools (Javkar, 2022).

*PRAWNS* scales to a large number of genomes, and generates a concise set of features that are comparable in number with those produced by whole-genome multiple alignment approaches, at a substantially lower computational cost. When applied to real biological data sets, *PRAWNS*’ features can be effectively employed to gain insights into phenotype associations as we have seen in examining antibiotic resistance in *A.baumannii*, where we have recapitulated known resistance determinants and also revealed new potential associations (Javkar *et al.*, 2021).

Currently, *PRAWNS* does not explicitly consider SNPs and short insertion/deletion events; however, the metablock structure can be used as an anchor for constructing multiple alignments of genomic regions in order to identify such events, and we plan to implement such functionality in future versions of our code. Like many other tools, *PRAWNS* does not handle variation within repetitive regions of a genome. The ability to handle pairwise interactions between conserved genomic elements may provide an opportunity for us to define a unique context for each repeated genomic segment, thus potentially resolving this long-standing problem in multiple-genome analysis. In its current implementation, *PRAWNS* relies on a straight-forward implementation of *k*-mers, which allows ample opportunity for refinement in order to improve performance, e.g. using minimizers or other hash-based techniques, as well as ideas from compressed de Bruijn graphs.

Overall, *PRAWNS* provides an efficient and effective framework for exploring genomic variations in large numbers of bacterial genomes, and we expect this tool to be a valuable part of the tool-kit used by scientists to analyze the rapidly increasing volumes of genomic data.

## Supplementary Material

btac844_Supplementary_Data
