# Supplementary material for: PRAWNS: compact pan-genomic features for whole-genome population genomics
Source: Bioinformatics. 2022 Dec 29;39(1):btac844. doi: 10.1093/bioinformatics/btac844 (PMC9825322; doi:10.1093/bioinformatics/btac844)
Supplement: btac844_Supplementary_Data [file btac844_supplementary_data.zip › PRAWNS_Supplementary_Material_final.pdf]

Supplementary Material

PRAWNS: Pan-genome representation of a large number of whole genomes

Kiran Javkar, Hugh Rand, Errol Strain, Mihai Pop

1. Motivation for finding metablocks by first identifying the components of collocated blocks:

Consider the following toy example where we have five genomes (G_1 to G_5). From these five genomes, say we have seven blocks (exact matching regions) (b_1 to b_7) identified as shown in the following figure:

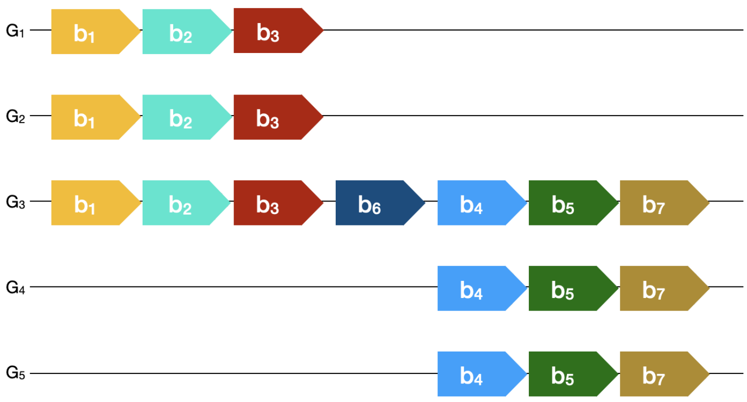

From the above figure, we can infer that the region bounding the blocks b_1, b_2, and b_3 is likely to similar and shared between the genomes G_1, G_2 and G_3. Likewise, the region bounding the blocks b_4, b_5, and b_7 is likely to be similar and shared between the genomes G_3, G_4 and G_5.

An important observation here is that the we get a much higher count of the exact matching regions (stemming from a variety of genomic variations, including SNPs and small insertions/deletions). It is desirable to merge and extend the exact matching regions to get a reduced count for the total number of genomic regions.

To merge and extend these exact matching blocks, we would like to identify the blocks co-occuring with a consistent relative orientations. For this, PRAWNS relies on the construction of a K-nearest neighbor (KNN) graph (Algorithm 1).
For the above example, a KNN graph (K=2) can comprise all seven blocks as follows:

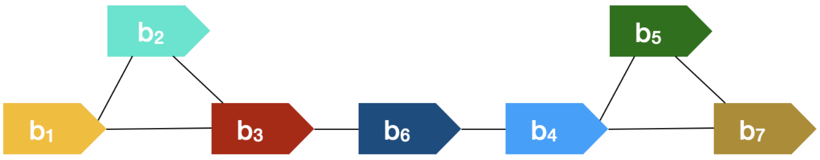

However, taking into account the collocation of these blocks, we will like to prune the above KNN graph to obtain two distinct groups or clusters of co-occuring blocks, shown as follows:

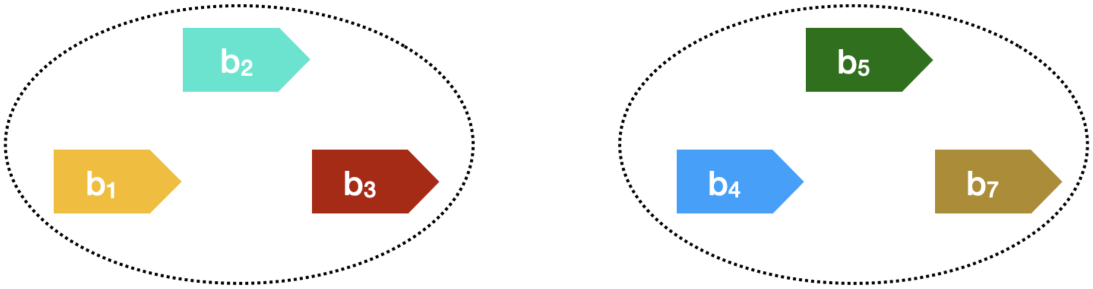

We refer to these clusters of blocks as the “components of collocated blocks”.

Once we obtain these components, we can merge the constituent blocks into a longer bounding regions which may contain inexact matches. Observe that some of these blocks may be collocated in some genomes but not have the same relative orientations as in the case of other genomes. It is recommended to have similar relative orientations of the constituent blocks for a non-ambiguous merge-and-extend action to get longer shared region detection. Algorithm 2 outlines the steps for identifying the blocks from a component of collocated blocks which can be merged into the “metablocks” and determining the set of genomes containing the same.

For the above example, the corresponding metablocks would be follows:

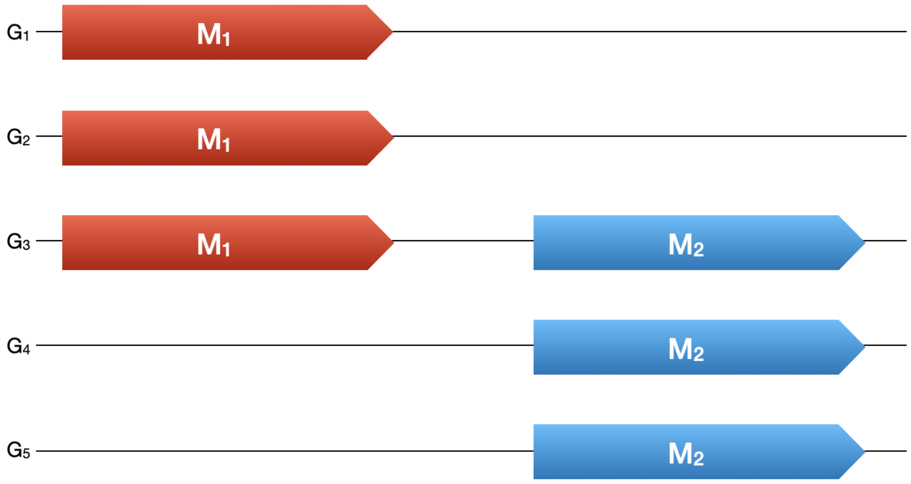

The metablock construction would provide a reduction in the feature count than the blocks alone, while ensuring that the genomic regions marked as the same metablock will be largely similar (may contain a few mismatches or indels depending on the separation between adjoining blocks—which is a user-defined parameter)

2. Connected component identification and Metablock detection

a. We begin with the construction of the K-nearest neighbor (KNN) graph. The graph construction is explained in Algorithm 1 and shown in Fig. 2:

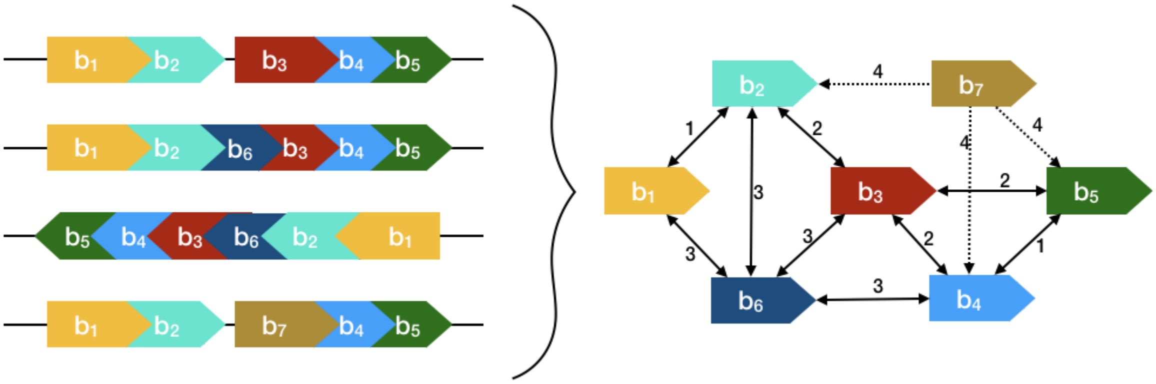

b. Next, we remove the edges between the vertices that do not have bi-directed edges. In other words, we retain only the bi-directed edges (solid edges) and remove the uni-directed edges (dotted edges) from the figure above.
The retained bidirected edges are replaced with undirected edges and run a minimum spanning tree algorithm to get the connected components. The minimum spanning tree for the above KNN graph would be as follows:

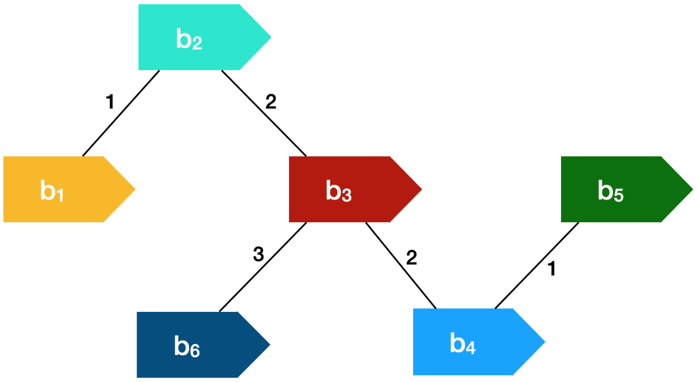

c. Each connected component within the "forest" of minimum spanning trees is referred to as a 'component of collocated *blocks*'. The presence of each component within a genome is empirically determined by using the presence of the constituent *blocks* of that component, as described in Algorithm 2.
Based on the empirical thresholds, the six blocks from the above figures could yield a component that is present in all four genomes or a component present in the top three genomes and not the last one.

d. As described in Algorithm 2, once the presence of a component has been established, we check for '*chains* of core *block* pairs'. Depending on whether the component was deemed present in all four genomes or the top three out of the four genomes from above, we would get the following sets of chains.

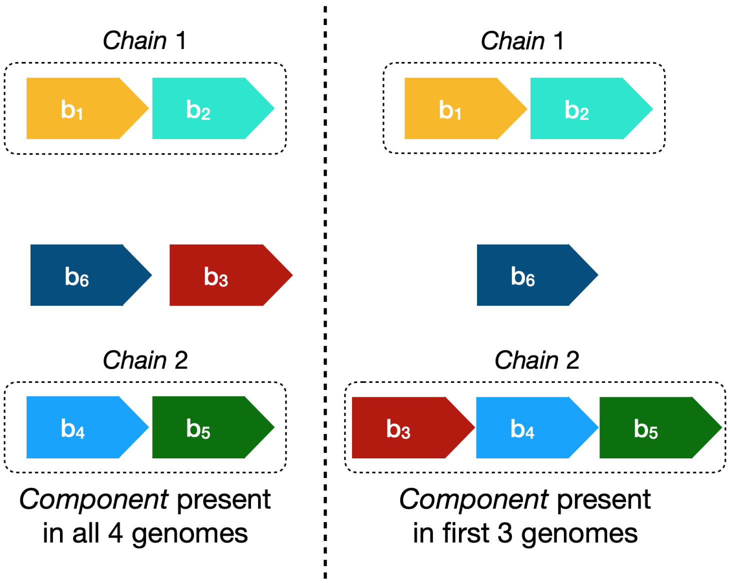

e. We follow the procedure from Algorithm 2 to deduce the *metablocks*. Depending on the deemed component presence and the value for the threshold μ, we can get the following different constructions for the *metablocks*.

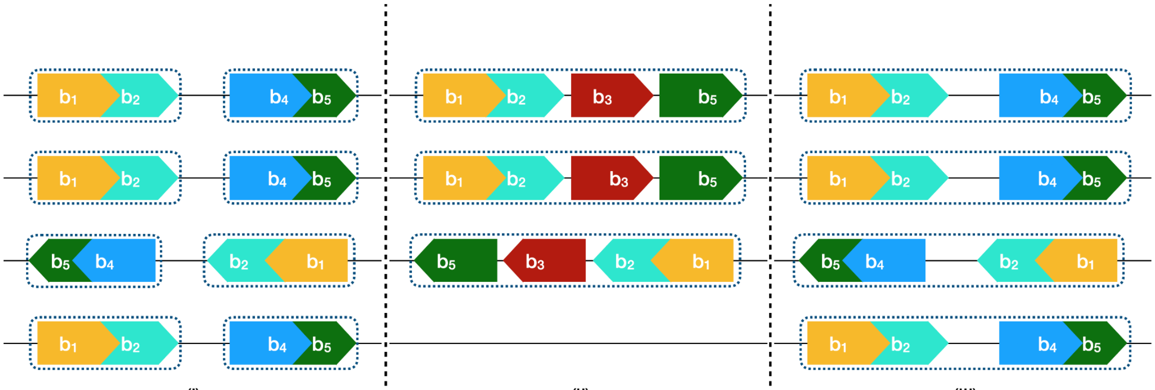

(I)
Component present in all 4 genomes:
2 Metablocks generated
present in all 4 genomes

(II)
Component present in 3 genomes:
1 Metablock generated
present in 3 genomes

(III)
Component present in all 4 genomes:
larger μ: 1 Metablock generated
present in all 4 genomes

f. Lastly, here's an instance of the impact of the threshold φ which is used for pruning the KNN graph.

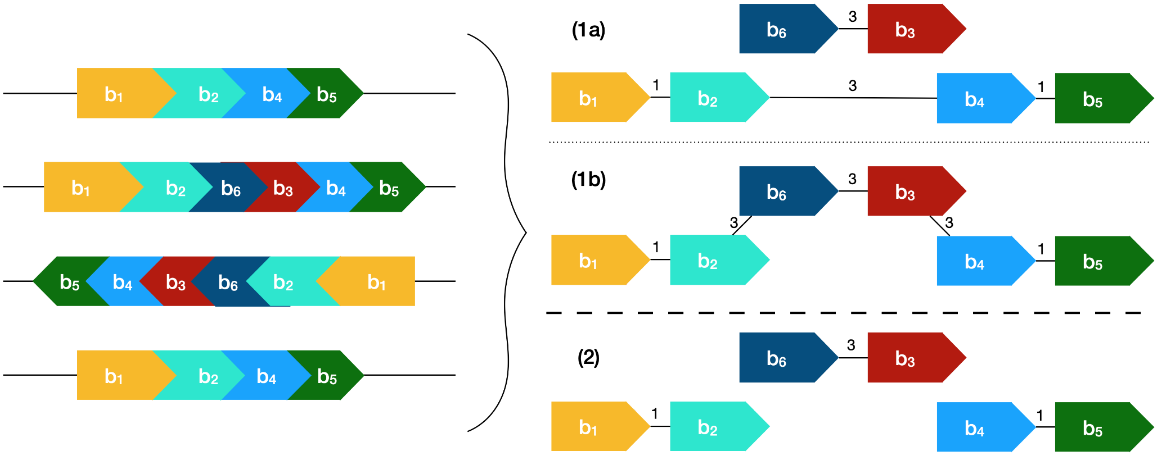

A higher φ (i.e. lower 1-φ threshold) means more neighbors and would result in either of the cases 1a or 1b, whereas a lower φ (i.e. higher 1-φ) would generate smaller connected components, like in case 2.
With case 1a, the component with four *blocks* may get split into two chains that end up as two *metablocks*, and one *metablock* from the other smaller component. (unless the chains are allowed to extend over larger separation (high δ) ⇒ redundancy and anomaly in *metablock* regions).
In case 1b, the single component may get split into two chains: b1–b2 and b4–b5. Like with 1a, either they may remain separate metablocks or get merged into a single *metablock*, based on the choice of δ. If they remain two *metablocks*, then using the paired regions features, we can observe that these *metablocks* have different separations, which may be important for certain analyses.
With case 2, we are would get three separate metablocks coming from three distinct components; the paired regions would encapsulate the separation differences as you would observe from case 1b.

3. Performance on longer (fungal) genomes

   a. To assess the scalability of PRAWNS on longer (eukaryotic) genomes, we ran it on a fungal dataset comprising 107 *Aspergillus flavus* genomes downloaded from NCBI. *A. flavus* contains 8 chromosomes and the genomes are approximately 38 Mbp each.
   b. Using the default *k*-mer length (25) on five cores each of 2.70 GHz Intel Xeon E5-2680 processor with 50GB maximum RAM usage limit, PRAWNS required 20 hours (40.26 GB peak memory usage) to generate the pan-genome comprising 858,081 conserved regions (427,698 *metablocks* and 430,383 retained *blocks* (γ=50)) and 1,020,418 paired regions (Δ=50).

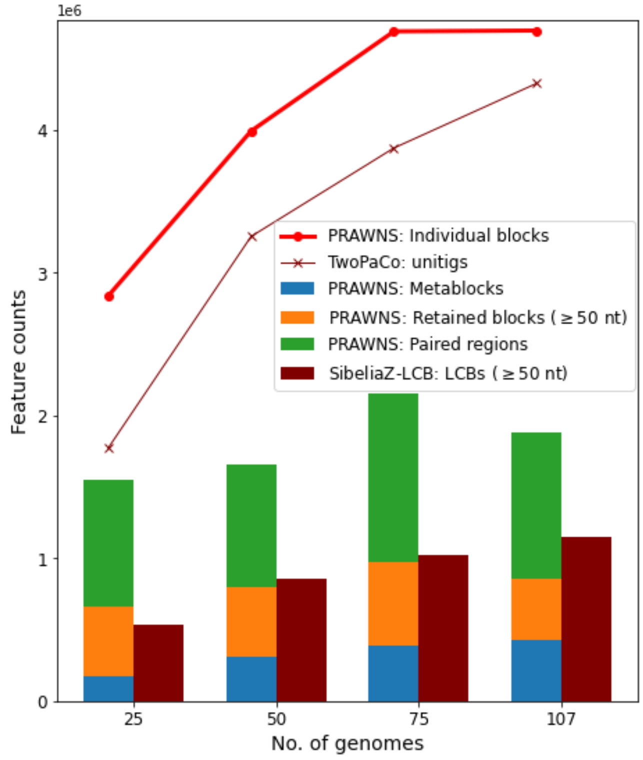

(a) Pan-genome feature counts

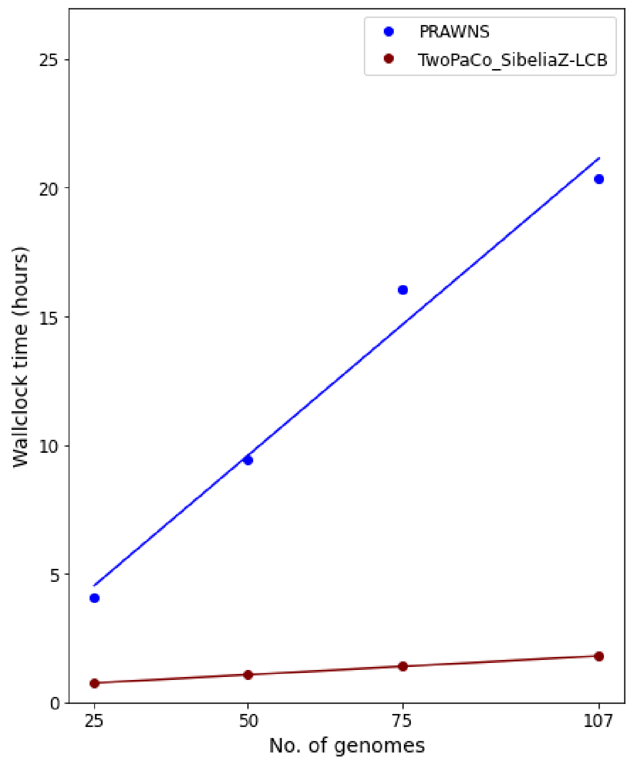

(b) Run-time

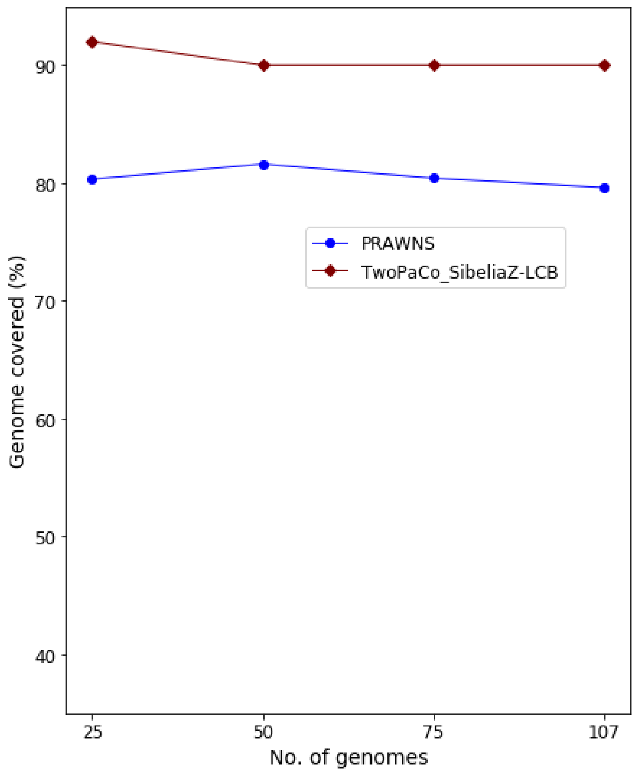

(c) Genome coverage

4. Empirical counts for components of collocated blocks

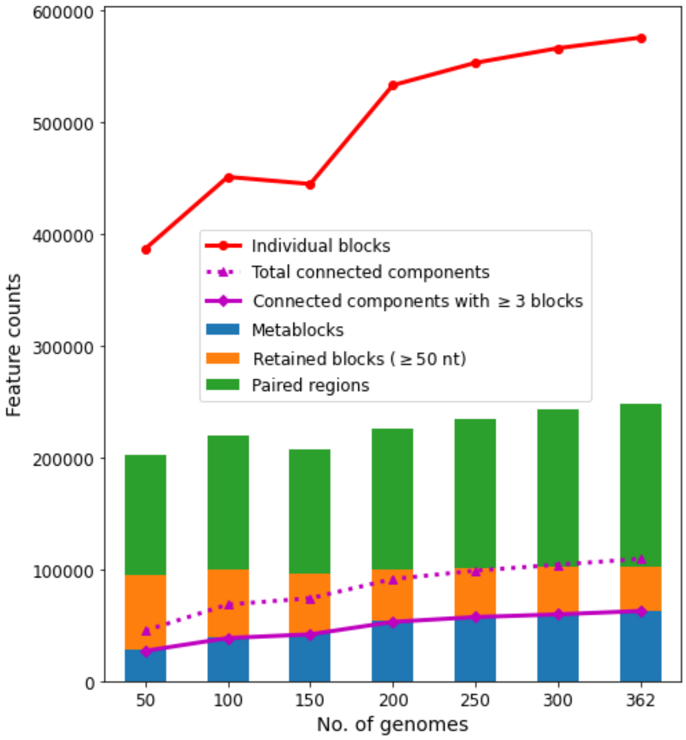

(a) *A. baumannii* dataset with results corresponding to Fig. 3

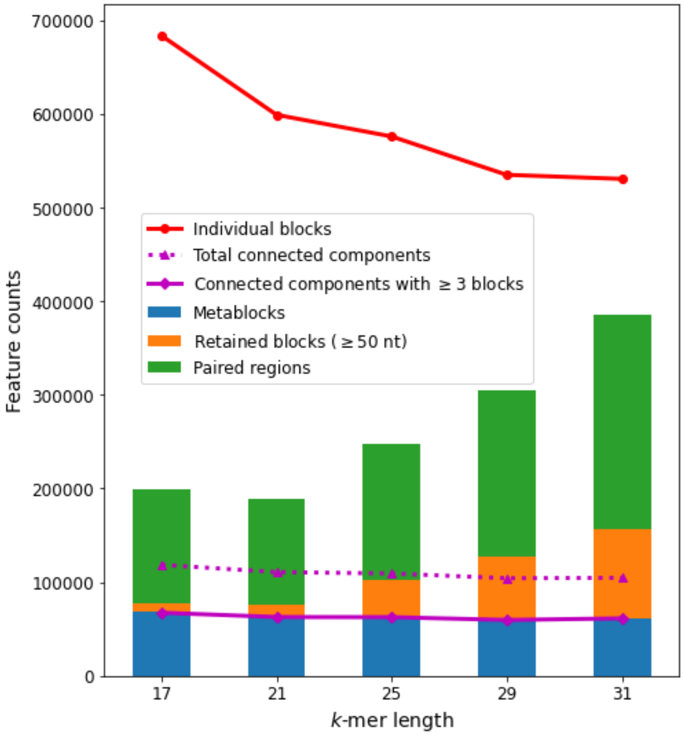

(b) *A. baumannii* dataset with results corresponding to Fig. 5

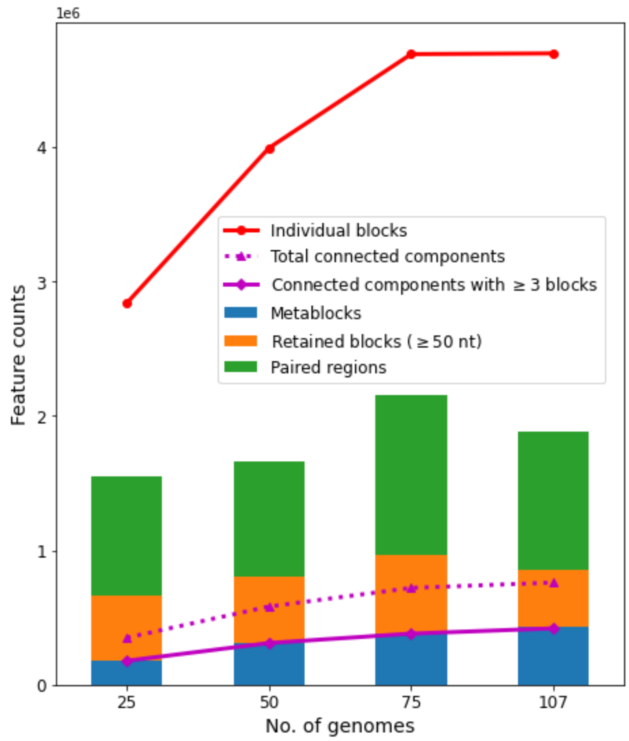

(c) *A. flavus* dataset with results corresponding to Section 2 of this Supplementary Material

5. Redundancy within LCBs from SibeliaZ-LCB

As shown in Fig. 4, the LCBs from SibeliaZ-LCB suffer from redundancy in the genomic regions identified under multiple LCBs.
To visualize this better, the following is an instance of two LCBs detected by SibeliaZ-LCB from the *A. baumannii* dataset (362 genomes); the conserved regions from PRAWNS which aligned to these LCBs are shown above the LCBs. The alignment was performed using BLAST and required at least a 90% sequence identity match.

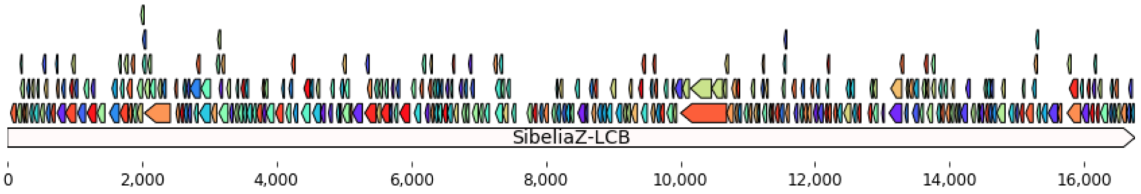

319 conserved regions (PRAWNS) aligned to LCB (SibeliaZ-LCB, id: 134848) of length 16,754 bp.

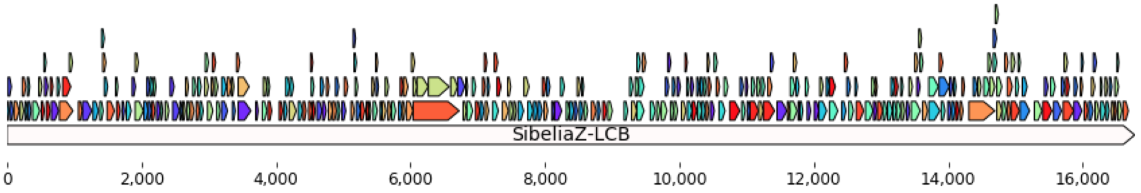

320 conserved regions (PRAWNS) aligned to LCB (SibeliaZ-LCB, id: 135813) of length 16,772 bp.

The above two LCBs essentially span over the same region. Additionally, from the variable colors assigned to the aligned conserved regions, it is evident that the constituent genomic regions have different memberships across the given genomes, and would be incorrect to group this into a single homologous region.
